# Flaxseed Extracts Impact the Cellular Structure of a Keratinocyte Model for Oral Lichen Planus—A Preliminary Study

**DOI:** 10.3390/ijms26125462

**Published:** 2025-06-06

**Authors:** Irena Duś-Ilnicka, Anna Prescha, Amanda Mordal, Kamila Środa-Pomianek, Beata Sobieszczańska, Monika Bielecka, Żaneta Czyżnikowska, Jakub Szperlik, Adam Matkowski, Małgorzata Radwan-Oczko

**Affiliations:** 1 Oral Pathology Department, Faculty of Dentistry, Wroclaw Medical University, Krakowska 26, 50-425 Wroclaw, Poland; malgorzata.radwan-oczko@umw.edu.pl; 2 Department of Dietetics and Bromatology, Wroclaw Medical University, Borowska 211, 50-556 Wroclaw, Poland; anna.prescha@umw.edu.pl; 3 Alumnus of the Faculty of Pharmacy, Wroclaw Medical University, 50-556 Wroclaw, Poland; amandamordal@gmail.com; 4 Department of Biophysics and Neurobiology, Wroclaw Medical University, ul. T. Chałubińskiego 3a, 50-368 Wrocław, Poland; kamila.sroda-pomianek@umw.edu.pl; 5 Department of Microbiology, Wroclaw Medical University, ul. T. Chałubińskiego 4, 50-368 Wrocław, Poland; beata.sobieszczanska@umw.edu.pl; 6 Department of Pharmaceutical Biology and Biotechnology, Wroclaw Medical University, Borowska 211A, 50-556 Wrocław, Poland; monika.bielecka@umw.edu.pl (M.B.); bbsekret@umw.edu.pl (A.M.); 7 Department of Basic Chemical Sciences, Faculty of Pharmacy, Wroclaw Medical University, Borowska 211a, 50-556 Wroclaw, Poland; zaneta.czyznikowska@umw.edu.pl; 8 Faculty of Biotechnology, Wroclaw University, Przybyszewskiego 63/77, 51-148 Wrocław, Poland; jakub.szperlik@uwr.edu.pl

**Keywords:** flax, lichen planus, oral, keratinocytes, oral potentially malignant disorders

## Abstract

Oral inflammation and the immune response are distinct but related processes where *Linum usitatissimum* L., fam. Linaceae represents a possible use for localized relief. Oral lichen planus (OLP) is an oral potentially malignant disorder (OPMD) with an inflammatory background that mainly affects post- and peri-menopausal women. The presented methodology was threefold. Firstly, the plant extracts were made from flaxseeds of selected cultivars (Szafir [SZ] and Jantarol [JA]) containing plant lignans. In silico docking affinity was performed to verify the beta and alpha estrogen receptors of keratinocytes’ (ERα and ERβ) affinity for lignans from the plant extracts. Lastly, tests using living keratinocyte cell lines were performed. Adding the studied extracts from two cultivars of flaxseed—JA and SZ (10 µg/mL) reduced lipopolysaccharides (LPS)—induced cell inflammation markers levels of COX-2 and IL-6. The effect of JA was more pronounced than that of SZ, with statistical significance (*p* < 0.05). A high in silico affinity was provided during secoisolariciresinol diglucoside (SDG) docking to ERα and ERβ. Flaxseed’s action could be based on the docking affinity of its major components to the estrogen receptors and the overall concentration of the elements of the extracts.

## 1. Introduction

Oral mucosa lichen planus (OLP) is the counterpart of cutaneous lichen planus [1]. This disorder mainly affects women in the fifth decade of life [2]. Clinically, it can have different presentations of white, red-white, erosive, and bullous lesions of various grades of extent and severity [3]. The course of this inflammatory disease is chronic, with recurring exacerbations [4]. The mechanisms of the T cell accumulation in lamina propria, disruption of the basement membrane, and keratinocyte apoptosis are described as the foundation of OLP development [5].

Because the etiology and the trigger factor in OLP pathogenesis are still unknown, the treatment available to the patients is mainly topical ointments for the pathologically changed oral mucosa to reduce the symptoms of active lesions [6,7,8]. The clinical state of the oral cavity of patients undergoing OLP is complicated by various accompanying symptoms, such as xerostomia, burning mouth symptoms, and pain or discomfort in the oral cavity [9,10].

The incidence of oral lichen planus is higher in perimenopausal women (10.91%) than in premenopausal women (0.5–2.0%) [2]. This may be related to the drop in estrogen levels during menopause, which has serious consequences for oral health [11]. Furthermore, expressed in keratinocytes, vesicles, and conducting tubules of the salivary glands [12], alpha and beta estrogen receptors (ERα and ERβ) [13,14] play a key role in regulating numerous complex physiological processes [15]. Inflammation and immune response are distinct but related processes observed in the oral cavity that protect it from infections and damage [16]. Inflammation is the localized response to tissue injury, infections, toxins, or other harmful stimuli. This response is characterized by symptoms such as redness, swelling, pain, and an increased temperature at the affected site [17]. The interaction between inflammation and the immune response is a significant reason for utilizing the human oral keratinocyte (HOK) model in OLP studies.

*Linum usitatissimum* L. (fam. Linaceae, linseed) consumption is commonly used to alleviate various ailments, including circulatory, respiratory, urinary, and digestive systems, or skin irritations [18]. In dentistry, there is an economical, plant-derived saliva substitute [19,20] accepted in clinical practice for dry mouth feeling, but the mechanism of its effectiveness is not clearly understood. It is suggested that the success of the therapy is influenced by the presence of plant mucus in flaxseeds, which might imitate the consistency of human saliva and cover the mucosa with an additional protective layer [21]. Flaxseed action on the oral mucosa has already been partially investigated by the effect of a salivary substitute prepared from flaxseed on relieving primary burning mouth syndrome and oral lichen planus [22,23]. However, it has not been investigated whether the similarity of rheological properties between flaxseed macerate and human saliva is the only factor influencing the improvement of the health of patients with OLP using flaxseed therapy [22] and if other forms of the herbal treatment based on it might be of use based on the potential molecular mechanism of its affinity.

Linseed contains many phytochemical compounds with potential clinical significance [24]. Among the plant phenolic compounds, lignans are classified as phytoestrogens, which in the human body demonstrate hormone-like activity by binding to previously described ERs [13,25]. Flaxseeds, as part of the plant, contain significant amounts of secoisolariciresinol (SECO), mainly in the form of diglycone (SDG) [26]. It has been found that SDG exhibits a wide range of biological activities, including antioxidant activity [27,28]. In addition to SECO, linseed contains other lignans, such as matairesinol and lariciresinol [29]. All these compounds are considered precursors for human microbiota metabolites exhibiting estrogenic activity. Screening studies conducted include molecular docking using the MolDock program, including SECO, lariciresinol, and pinoresinol, demonstrating the compounds’ affinity for ERα and ERβ. However, no in silico docking analyses regarding SDGs and ERs have been published [30]. Given the clinical relevance of flaxseed gel that is used as a saliva substitute and its potential affinity to the ERα and ERβ receptors, we considered it relevant to evaluate the molecular mechanism underlying the clinically observed positive outcome of the therapy described above—to understand the potential to alleviate the shortcomings of OLP in perimenopausal women.

This preliminary study aimed to evaluate the phenolic composition of methanol extracts from two cultivars of linseed, Jantarol and Szafir, and assess their ability to inhibit inflammation induced in keratinocytes in vitro. In addition, the binding capacity to the ER receptors of SDG, which is the main constituent of the polyphenolic extract, was evaluated by in silico docking.

## 2. Results

### 2.1. Concentration of Polyphenols in Szafir and Jantarol Extracts

UPLC analysis of plant extracts obtained from the seeds of common flax cultivars Szafir (SZ) and Jantarol (JA) confirmed the presence of SDG in both samples. The spectra obtained during the analysis also confirm the presence of other phenolic compounds in the extracts tested. The analysis of the spectra (Supplementary Table S1) showed the presence of caffeic acid glucoside, *p*-coumaric acid glucoside, ferulic acid glucosides, *p*-coumaric-ferulic glucoside, caffeic-*p*-coumaric glucoside, *p*-coumaric acid, and ferulic acid (Table 1).

### 2.2. Binding to the Alpha and Beta Estrogen Receptor—Molecular Docking Study

**Figure 1:**
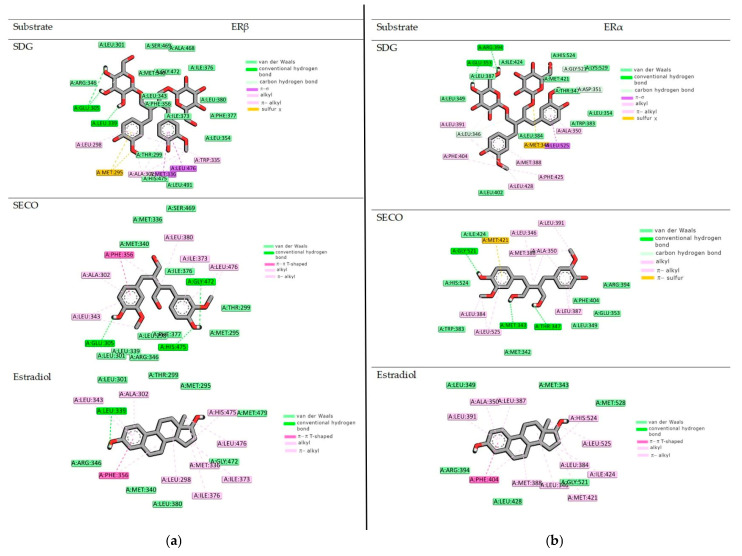
Graphical representation of the intermolecular interactions at the receptor binding site. (**a**) ERα and (**b**) ERβ.

Because bacteria do not metabolize SGD and SECO to active derivatives, we investigated the affinity of the primary compound from the lignan group, SGD. In the present study, the binding mode of two SECO and SGD to the ligand binding pocket of both ERα and ERβ was predicted using a molecular docking protocol and compared with the position of estradiol (Figure 1). The docking procedure was validated by redocking estradiol to the crystal structure of both receptors. The results obtained for the docking accuracy had an RMSD of less than 0.75. Aware that scoring functions used in the docking algorithms give only approximate values of free energy of binding, the results were validated with biological activity measurements, which will be carried out in further research. The obtained data can be found in Table 2a,b.

The binding affinity of ligands estimated during molecular docking simulation is related to the Gibbs energy of binding and is expressed by the following formula:

ΔGbind = [ΔG intermolecular + ΔG internal + ΔG tors] − ΔG unbound(1)

Intermolecular interaction energy (ΔEint) is the sum of van der Waals, hydrogen bonding, desolvation, and electrostatic terms between the inhibitor and the protein’s binding site.

ΔEint = [ΔEvdw + ΔEhbond + ΔEdesolv] + ΔEel(2)

Therefore, the binding potency of the investigated ligand depends on the mutual relations of all sources of stabilization of the obtained complex. According to data from calculations, both compounds SDG and SECO can bind to the binding center of ERα and ERβ. The differences in the binding potency estimated during the docking study were not crucial. For example, both considered compounds bind to the ERβ with the same free energy value of binding (about −11 kcal mol). However, it is essential to note that the reported binding energies for SDG, SECO, and estradiol differ by only 1–2 kcal/mol, which falls within the typical margin of error for molecular docking methods. Therefore, these slight differences should not be taken as definitive evidence of binding selectivity.

**Figure 2:**
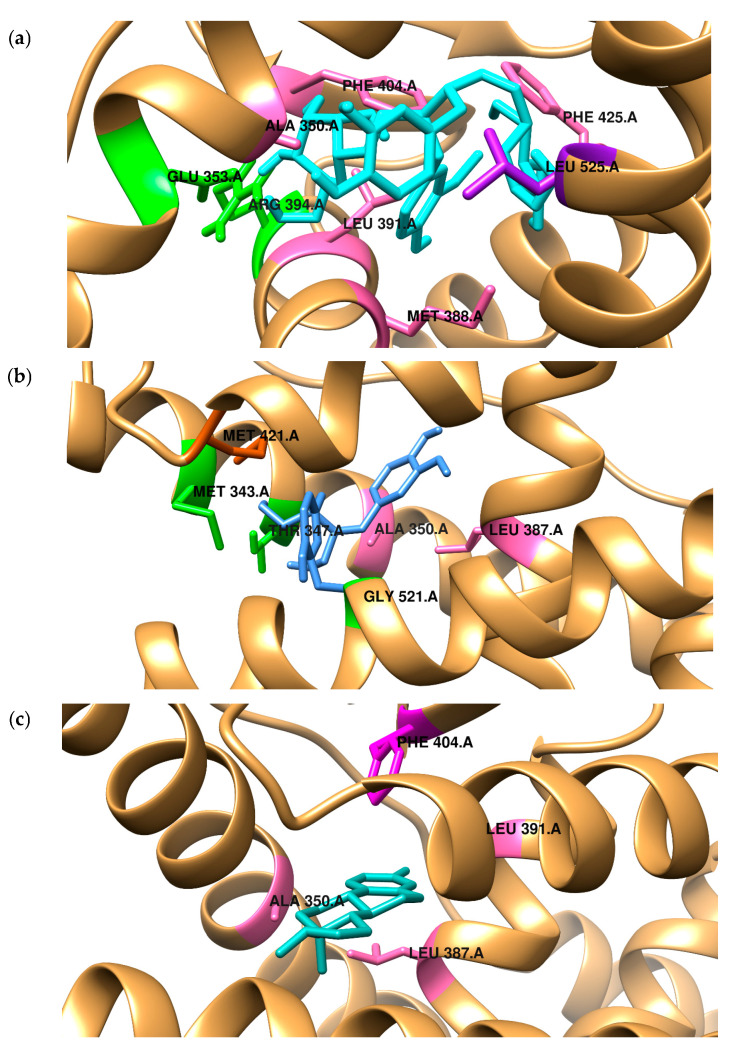
The comparison of the binding mode of (**a**) SDG (**top**) and (**b**) seco (**middle**) with the position of (**c**) estradiol (**bottom**) in the binding pocket of ERα. Intermolecular interactions are marked by hydrogen bonds—green, π-alkyl—pink, π-σ—purple, π-sulfur—orange, π—π—T-shaped—magenta.

**Figure 3:**
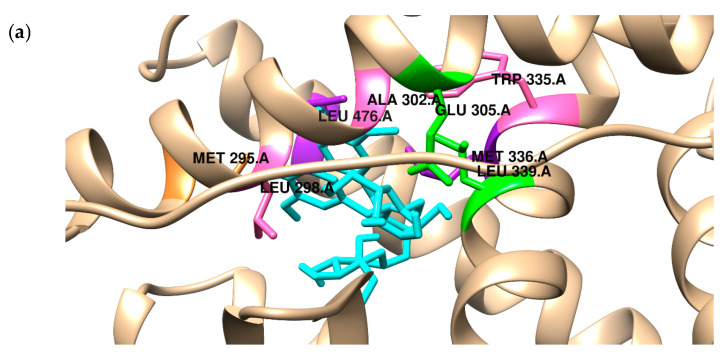

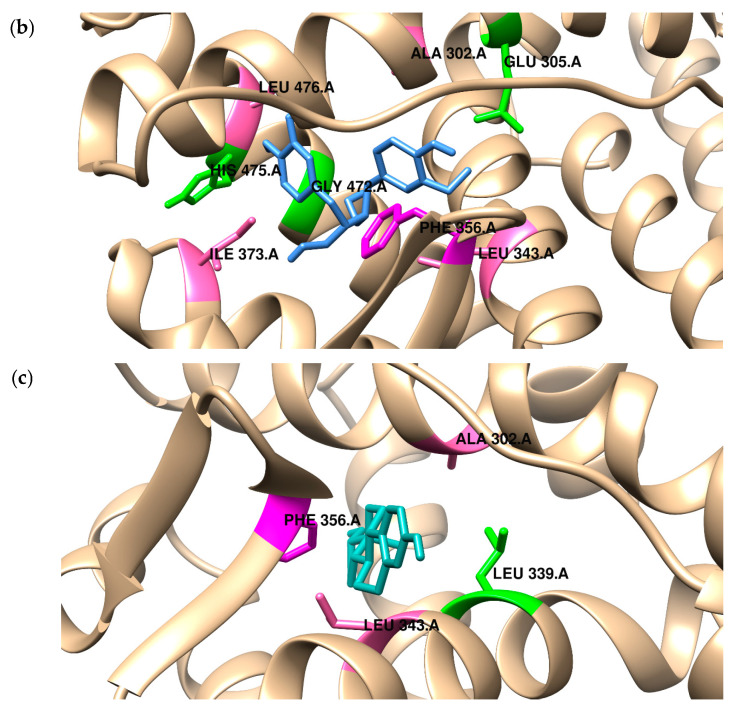
The comparison of the binding mode of (**a**) SDG (**top**) and (**b**) SECO (**middle**) with the position of (**c**) estradiol (**bottom**) in the binding pocket of ERβ. Intermolecular interactions are marked by: hydrogen bonds—green, π-alkyl—pink, π-σ—purple, π-sulfur—orange, π—π—T—shaped magenta.

Additionally, in Table 2, we present the quantitative characterization of the stabilization sources of the complexes formed. In all considered cases, the origins of stabilization are non-covalent interactions, mainly hydrophobic terms, but also hydrogen bonds and van der Waals interactions (see the value of ΔE1). Electrostatic interactions do not play a role in stabilization. Figure 1, Figure 2 and Figure 3 visualize the binding modes of compounds and origins of stabilization according to their type.

Although our docking results suggest that SDG and SECO may interact with ERα and ERβ through similar non-covalent forces as known estrogen receptor modulators, it is essential to underline that these results are purely theoretical. No experimental validation of receptor modulation has been performed, such as binding affinity studies. Therefore, the proposed binding method should be considered a hypothesis that requires further investigation.

### 2.3. Analysis of the Effect of Flaxseed Extracts on the Keratinocytes

To investigate the effect of Jantar and Szafir on OLP, HOK cells were treated with LPS (endotoxin) to mimic the state of infection-induced OLP, and then IL-6 and COX-2 levels were assessed. The treatment of HOK cells by LPS induced an inflammatory response, resulting in a substantial increase in COX-2 and IL-6 produced by the cells (A and B, respectively, in Figure 2) [31,32].

**Figure 4:**
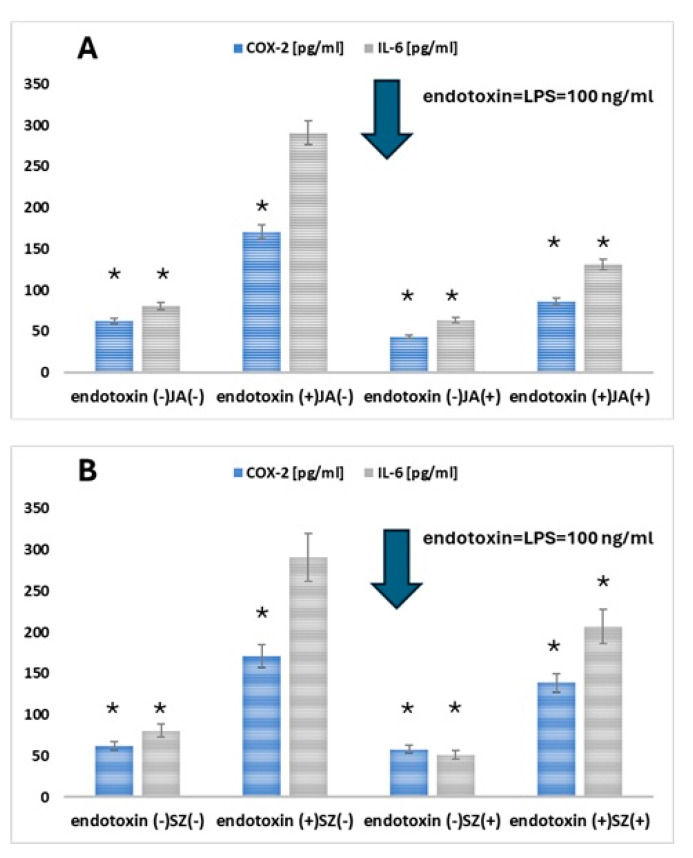
COX–2 and IL-6 expression in keratinocytes after adding the studied flax cultivars (Jantarol (JA) (**A**) and Szafir (SZ) (**B**)) (10 µg/mL) to the keratinocyte cell lines reduced LPS-induced levels of both inflammation markers. The means of three experiments ± SD are presented. The statistically significant differences from the untreated HOK control were determined using Student’s *t*-test (* *p* < 0.05).

Adding the extracts of studied cultivars (JA, SZ) (10 µg/mL) reduced LPS-induced levels of both inflammation markers. The effect of JA was more pronounced than that of SZ (statistical significance (*p* < 0.05) and was obtained both in the case of COX-2 and IL-6 (Figure 4).

It was demonstrated that both studied compounds could modulate the inflammatory response of HOK cells. To avoid confounding the interpretation of anti-inflammatory effects with potential cytotoxicity, we chose a 10 µg/mL concentration, which allows for a clear assessment of the anti-inflammatory properties while minimizing any cytotoxic influence on HOK cells.

The observation that plant-derived compounds or extracts can exert both anti-inflammatory and pro-inflammatory effects depending on their concentration is a well-recognized phenomenon and can be partly explained by the hormesis concept. Hormesis describes a biphasic dose–response relationship in which a compound elicits beneficial effects at low doses but becomes harmful and cytotoxic at higher concentrations.

**Figure 5:**
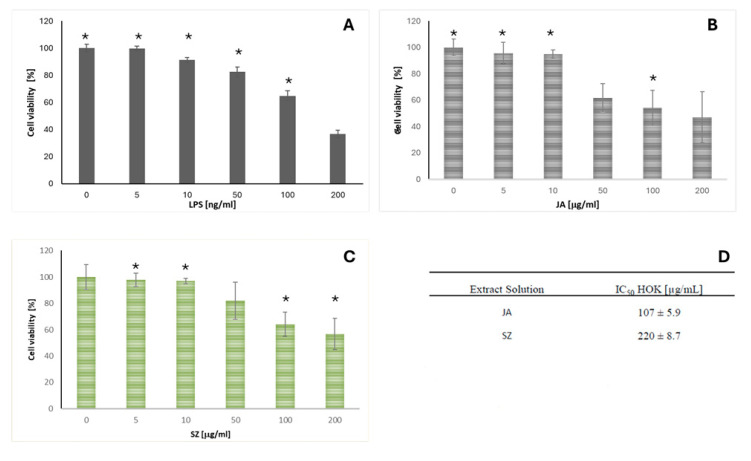
HOK cells treated with LPS (100 ng/mL) to mimic the OLP (oral lichen planus) state induced by infection (**A**). The influence of two studied cultivars (Szafir (SZ) and Jantarol (JA)) on HOK cell viability was assessed. Both extract solutions were revealed to possess cytotoxic activity against HOK cells (**B**,**C**), as shown by the results of the MTT assay—the half maximal inhibitory concentrations (IC50) of the studied extract solutions in HOK cells (**D**). The means of three experiments ± SD are presented. The statistically significant differences from the untreated HOK control were determined using Student’s *t*-test (* *p* < 0.05).

Therefore, the influence of both extract solutions on HOK cell viability was also assessed. The selection of this specific concentration range was based on preliminary studies. The studied flaxseed cultivars (JA, SZ) exhibited cytotoxic activity against HOK cells (Figure 5B,C). JA exhibited greater activity than SZ. The IC50 values obtained for JA and SZ were 107 ± 5.9 µg/mL and 220 ± 8.7 µg/mL, respectively. The most cytotoxic concentration identified in the dose–response experiments was also the IC50 value. Higher concentrations (230–1000 µg/mL) did not inhibit cell growth more than the identified IC50 value (additional data are provided in Figures S1 and S2 in the Supplementary Data).

## 3. Discussion

Commonly represented in female patients, OLP [33,34] is an OPMD with some risk of malignant transformation to oral squamous cell carcinoma [3,35]. These patients require constant medical and dental assistance, as accompanying disorders from the oral cavity affect them throughout their lives, with periods of relapses and remissions [36,37,38]. Because of a burning sensation, xerostomia, and pain of the changed oral mucosa, diverse medical products (sprays, ointments, and drops) are used in local treatment to relieve symptoms and facilitate the daily routine, like cleaning teeth or eating [10,38,39]. Especially in female perimenopausal patients with OLP, potential medicinal products are searched based on different approaches for estrogen deficiency and its related outcomes that are presented in the oral cavity. Eliasson et al. demonstrated the effect of low-concentration estrogen therapy on increasing saliva production by the salivary glands and reducing the number of patients complaining of dry mouth [40]. However, the oral mucosa is not considered a traditional target site for estrogens; the confirmed presence of ERs in both the oral mucosa and salivary glands suggests that these tissues may be sensitive to changes in estrogen levels [41].

Flaxseed is a rich source of omega-3 fatty acids, alpha-linolenic acid, SECO, and fiber. It benefits human health through anti-inflammatory action, anti-oxidative capacity, and lipid-modulating properties [42]. Lignans in the linseed are phenolic compounds with antioxidant properties, also classified as phytoestrogens [43]. Phytoestrogens, due to their structural similarity to female sex hormones—estrogens—can bind to human ERs and exhibit hormone-like effects [44,45]. To confirm whether the lignans can bind to the ERβ or ERα receptors on the keratinocytes, in silico docking has been performed in this study to establish whether this binding is possible. In recent years, estrogens’ role in regulating epidermal cells’ function, including keratinocytes, has gained significant attention in research concerning oral health. In the context of studies on keratinocyte metabolism, SECO emerges as an intriguing compound that may influence interactions between estrogens and the function of these cells. The literature suggests that SECO may interact with various signaling pathways, influencing keratinocyte proliferation, differentiation, and apoptosis [42,43,44]. Understanding how SECO can modulate its function through interactions with estrogen receptors is essential for developing new therapeutic strategies.

Modeling the impact of SECO on HOK keratinocytes in the context of estrogen receptor activation aims to investigate how this compound may influence metabolism and behavior in epidermal cells [44,45]. Incorporating SECO into our research seeks to elucidate its role concerning estrogen receptors and expand knowledge regarding its potential applications in treating conditions affecting the epidermis, such as dermatological diseases and inflammatory states. We believe that the results of our studies will contribute to a better understanding of the interactions between hormones and keratinocyte metabolism, opening new perspectives in regenerative medicine and dermatology.

ERs have been observed to bind a range of molecules with various properties: agonist, mixed agonist–antagonist, and full agonist characteristics [46]. The compounds can bind to the ligand-binding domain, which exhibits 56% amino acid homology between the ERs [47]. Most crucial amino acids within the ligand-binding cavity responsible for binding antagonists or selective estrogen receptor modulators are almost identical. It was demonstrated that the ligand is stabilized by two main forces: hydrogen bonding and van der Waals interactions [48], as shown in our study. In the receptor ERα binding pocket, a specific residue, Glu353, can form hydrogen bonds. The conserved Arg394 also plays a unique role in the positioning of ligands. Another crucial structural motif is the π-contact of Phe404 with the hydrophobic estradiol ring. As illustrated in Figure 2a, the SDG compounds can form three hydrogen bonds with the receptor’s amino acid residues, Glu353 and Arg394. Similarly to estradiol, SDG is stabilized in the binding pocket by van der Waals and alkyl interactions with Leu349, Ala350, Met388, Met421, Leu428, and His542. The Phe404 residue contacts the ligand through a π-alkyl interaction. In contrast, three hydrogen bonds are created with Met343, Thr347, and Gly521 with SECO. The amino acid residues Glu363 and Arg394 interact with the ligand via van der Waals forces. The rest of the phenylalanine is distant from the ligand molecule and can only interact via van der Waals interactions. According to earlier studies, estradiol is deeply embedded in the ligand-binding domain of ERβ [49]. In this case, ligand stabilization is responsible for hydrophobic interactions with Met295, Leu298, Leu301, Met336, Met340, Leu343, Ile373, and Leu476. Both compounds considered in our study exhibit similar binding patterns. SDG compounds also interact hydrophilically with Glu305 and Leu305 amino acid residues. The π-type interactions stabilize the SECO conformation. In future studies, we plan to extend the in silico studies to include molecular dynamics simulations to predict the dynamic aspects of their binding to the receptor. However, we recognize that drawing functional conclusions solely from docking studies has limitations. Accordingly, our findings support a hypothetical binding mode that requires experimental confirmation. Although the docking results suggest potential interactions between the studied ligands and estrogen receptors, further biological studies, such as receptor binding, are necessary to verify their modulatory activity.

In silico docking in the therapy of the effects related to menopause can represent a high value, as shown by Amany and Priyanka [50,51], in understanding the molecular basis of the action. Those tests preceded the in vivo validation required for its clinical application. In the present study, we have implemented the first analysis using the HOK cell line, which finishes the first cycle of the research and represents a solid basis for further clinical study.

The tested flaxseed, Jantarol, and Szafir have the same profile of phenolic compounds but differ in their content. The primary phenolic compound in both cultivars—secoisolariciresinol diglucoside (SDG)—was measured at 50% in Szafir and 76% in the Jantarol cultivar of the flaxseed. Secondly, ferulic acid was expressed in a higher concentration in Szafir than in Jantarol. This phenolic substance, widely found in plants, is an essential active component and has beneficial biological activities against oxidative stress and inflammation, as discussed in this study on the keratinocyte cell lines [52]. All the represented variations, however, did not statistically influence the difference between the outcome of the keratinocyte’s cell culture, representing both extract solutions (JA and SZ), which were revealed to possess cytotoxic activity against HOK cells.

OLP is often associated with dysregulation of the immune response. Studies on HOK in the context of inflammation may contribute to a better understanding of the immunological mechanisms, which may lead to the development of new therapeutic strategies. The potential for therapy is in understanding the mechanisms that trigger inflammation in HOK, which may provide valuable clues to potential therapeutic targets for treating OLP, especially when using anti-inflammatory or immunomodulatory drugs. Inflammation plays a crucial role in maintaining health as a self-protective immune response. However, long-term chronic inflammation leads to immune dysregulation and accumulation of inflammatory cells in tissues. As we have presented, the enzyme COX-2 is required to produce prostaglandin E2 (PGE-2), which can cause severe pain in humans during inflammation. Hence, inhibition of COX-2 is key to alleviating inflammatory symptoms [53]. Compared to the control group, COX-2 levels in the LPS group were increased, while they were decreased in the presence of extracts from the JA and SZ cultivars. Adding the extracts of the studied cultivars (JA, SZ) reduced LPS-induced levels of both inflammation markers.

Many phytochemicals, such as polyphenols, flavonoids, and lignans (including those found in flaxseed), can act as antioxidants at sub-cytotoxic or low concentrations by scavenging reactive oxygen species (ROS), upregulating endogenous antioxidant defenses (e.g., the Nrf2 pathway), and modulating redox-sensitive signaling pathways. These effects contribute to their anti-inflammatory and cytoprotective properties. However, as the concentration increases, these compounds may disrupt essential cellular functions, such as membrane integrity, mitochondrial activity, or DNA replication, leading to cytotoxic outcomes. This is especially relevant for compounds with antioxidant or anti-inflammatory properties, which may become pro-oxidant or harmful at higher doses.

This concentration-dependent duality is well-documented in pharmacology and toxicology and highlights the importance of identifying an appropriate therapeutic window where the compound is effective but not toxic.

Both studied flax cultivars (JA and SZ) differ in their phenolic composition. Still, the overall anti-inflammatory effects observed at the tested concentration (10 µg/mL) were comparable, with JA showing a slightly more pronounced effect. This apparent discrepancy can be explained by several factors related to the functional relevance of individual phenolic compounds and the complexity of their biological activity. Firstly, it is essential to note that biological effects are not always directly proportional to the total phenolic content or the presence of specific compounds in isolation. The bioactivity of plant extracts often results from synergistic or additive interactions between multiple components, including minor constituents that may have high biological potency. Thus, even if the profiles differ qualitatively or quantitatively, the overall functional outcome, such as anti-inflammatory activity, may appear similar due to these interactions. Secondly, individual phenolics’ bioavailability, cellular uptake, and metabolic stability can significantly influence their biological effects. Some compounds may be present in higher amounts in one cultivar but exhibit lower activity due to limited cellular accessibility or rapid metabolism. Conversely, a cultivar with a lower total phenolic content may contain more readily bioactive compounds at the cellular level.

Moreover, in the present study, we aimed to evaluate the extracts’ phenolic composition and assess the ER receptors of SDG via in silico docking. Those parameters establish the basis for the preliminary analysis of the fast-forward question: why does the topical use of flaxseed extracts bring relief to patients with oral mucosa inflammation? Previously provided studies included the effect of a homemade salivary substitute on oral mucosa with the use of prepared flax seed to provide relief of primary burning mouth syndrome and oral lichen planus [22,23,54]. Also, the discourse started about other possible and available herbal remedies that might alleviate the symptoms related to those oral disorders [23]. Given the importance of this topic for the patients affected by OLP, we find that this preliminary study sets the path for the following discourse in studying anti-inflammatory activity to provide new intervention strategies for alleviating oral mucosa disorders.

## 4. Materials and Methods

Firstly, the plant extracts were made from flaxseeds of selected cultivars (Szafir and Jantarol) containing plant lignans. Szafir is an oil flax cultivar harvested when the seeds are brown. Jantarol is one of the varieties of oil flax harvested when the seeds are intensely golden. Szafir and Jantarol were obtained from Plant Breeding Strzelce Sp. z o.o. IHAR Group from the Borowo branch in Czempiń, Poland. The seeds come from the 2020 harvest.

- Jantarol (breeding name of the cultivar BOH 505)—germination capacity (germ.): 72%, thousand-grain weight (MTZ): 7.4 g, 2020 harvest, seed color: yellow
- Szafir (breeding name of the cultivar BOH 191)—germ.: 80%, MTZ: 8.2 g, 2020 harvest, seed color: brown.

Jantarol was registered with COBOR (Research Centre for Cultivar Testing) Catalog Number BOH 505, Index Entry R 1006, and Szafir was registered under Catalog Number BOH 191 (K 80/58a-1446), Index Number R 170. Both cultivars of flax (*Linum usitatissimum* L.) were sourced from IHAR Strzelce (Hodowla Roślin Strzelce Sp. z o.o Grupa IHAR, Poland). The composition of the two cultivars was confirmed and analyzed using UPLC (Ultra-Performance Liquid Chromatography). Secondly, in silico docking was performed to verify the lignans’ affinity to ERα and ERβ of keratinocytes with the plant extracts. Furthermore, the plant extracts and data obtained during the project were the foundation for conducting cell culture in the laboratory using living keratinocyte cell lines.

### 4.1. In Silico Docking for the ERα and ERβ Affinity Methodology

The molecular docking was used to determine whether the lignans contained in linseed can potentially bind to ERβ located in oral tissues (specifically in the keratinocytes) and thus influence their expression. The structures of all compounds considered in calculations were optimized at the B3LYP/6-31++G** level of theory using the Gaussian 09 package [55]. The polarizable continuum model was adopted during optimization to consider the solvent effect [48]. AutoDock4.2 program and the standard protocol were used to predict the binding mode of compounds to the ligand binding pocket of both ERα and ERβ [56]. The receptor structures were downloaded from the Protein Data Bank. We used ERα (PDB ID: 1A52) and ERβ (PDB ID: 3ols) crystals co-crystallized with estradiol as a molecular target [57,58]. The docking procedure was validated by docking the estradiol molecule into the receptor and comparing its position with the crystal. The root mean square deviation (RMSD) was calculated to estimate the accuracy of docking prediction on the LigRMSD web server [59]. The binding mode of compounds was correctly predicted when their RMSD was less than 1 Å. Our previous studies detailed the protein and ligand preparation and docking procedure [60,61]. A Lamarckian genetic algorithm with local search was employed, and 200 runs were performed for the binding site [33]. Previous studies have shown that it is the most efficient and reliable AutoDock4.2 algorithm [34]. The obtained results were visualized using Chimera and a BIOVIA Discovery Studio visualizer (BIOVIA Discovery Studio. Discovery Studio Visualizer; BIOVIA Discovery Studio: San Diego, CA, USA, 2019).

### 4.2. Preparation of the Extracts from Szafir and Jantarol Cultivars

A total amount of 150 g/sample of raw flaxseeds of both types was used to de-fat the ground seeds, according to the methodology obtained from the manuscript authored by Zeitoun et al. 2014 [62]. Briefly, the sample flaxseeds were ground to obtain a fine powder. The samples were defatted by blending the ground materials with hexane (1:10 *w*/*v*) at 60 °C for 15 min, three times. The sediment was allowed to dry for 24 h in a fume hood, excluding the remaining hexane from the mixture. The dried, defatted seeds were weighed, and 92.15 g of Jantarol and 93.14 g of Szafir were obtained, constituting 61.4% and 62.09% of the initial samples, respectively. Then, the defatted powder obtained in triplicate was air-dried for 18 h at 37 °C and stored at −20 °C for later use. Extraction of phenolics was carried out for 15 min using 70% (*v*/*v*) methanol three times, each time using 400 mL of the reagent. The solution was then transferred to 500 mL glass bottles and subjected to alkaline hydrolysis for over 48 h. A total of 1 mL of previously prepared 0.3 M NaOH was used for hydrolysis. The obtained solution still contained residual sediment. Therefore, it was decided to vacuum filter using a Buchner funnel and a hard quantitative filter. The obtained filtrate was neutralized with 2 M HCl. This way, 340 mL of extract was obtained from 150 g of raw seeds of the Jantarol, and 305 mL of extract from 150 g of the Szafir cultivars. The extracts obtained were partially evaporated using a Speed Dry vacuum concentrator at 40 °C and 1300 rpm for 12 h. The samples were placed in the freeze dryer for 12 h, and then the drying process lasted 4 h at a pressure of 0.055 mbar. Using empirical method, a sufficient amount of solvent was determined to be 12 mL of deionized water for each sample. From each sample, 200 µL of the extract was collected in vials for analysis using UPLC. The rest of the prepared plant extracts containing flax lignans and other phenolic compounds were used in studies on cell lines.

### 4.3. Determination of SDG and Other Phenolic Compounds Content in Methanol Extracts of Phenolic Acids via Ultra-Performance Liquid Chromatography (UPLC)

Methanol extracts of phenolic acids from defatted seeds were subjected to chromatographic analyses for separation and quantification of phenolic compounds, according to Zeitoun et al. 2014 [62]. Chromatographic analysis was performed using a Waters Acquity UPLC ultra-performance liquid chromatograph. An Acquity UPLC BEH C18 column was used for the study. The injection volume was 5 µL. Elution was performed in a gradient manner using the following solvent mixtures: mixture A: 99.9% H_2_O/0.1% formic acid (*v*/*v*); mixture B: 100% acetonitrile (*v*/*v*). The flow rate of the mobile phase was 0.4 mL/min. The elution steps were as follows: 0–1 min isocratic separation at 10% B, 1–10 min linear gradient to 30% B, 10–12 min linear gradient to 100%, and a return to the initial conditions for an additional 1 min. The analysis was performed using an empirically determined eluent flow program.

**Figure 6:**
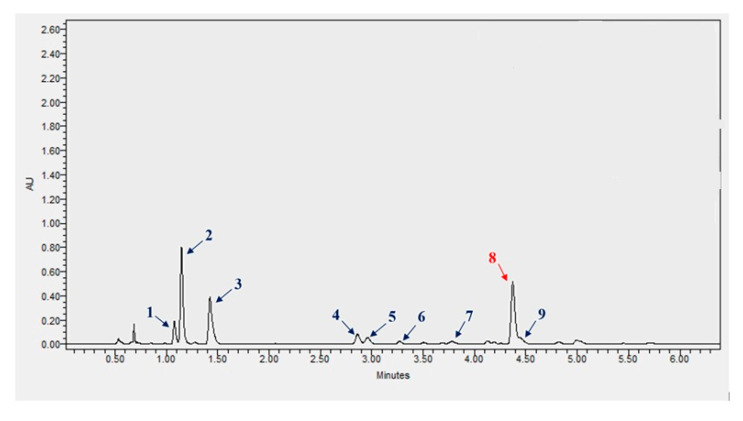
UPLC chromatogram showing the separation of phenolic compounds in flax seed extract, Jantarol cultivar. Legend: 1: caffeic acid glucoside, 2: *p*-coumaric acid glucoside, 3: ferulic acid glucoside (2), 4: *p*-coumaric-ferulic glucoside, 5: caffeic-*p*-coumaric glucoside, 6: ferulic acid glucoside (1), 7: *p*-coumaric acid, 8: SDG, 9: ferulic acid.

Chromatograms were recorded at wavelengths of 280 and 320 nm. Individual phenolic compounds’ peaks were identified by comparing their retention times (RT) and UV-VIS spectra with data obtained from the separation of standard compounds (Figure 6). The content of secoisolariciresinol glycoside in extracts from flaxseeds of JA and SZ cultivars was calculated based on the standard curve for secoisolariciresinol glycoside. The contents of other phenolic compounds were calculated based on the standard curves for caffeic acid, *p*-coumaric acid, and ferulic acid.

### 4.4. Cell Culture Cell Preparation

The treatment of Human Oral Keratinocyte (HOK) cells with lipopolysaccharides (LPS) and the simulation of inflammation as a model for oral lichen planus (OLP) were used in the present study for several reasons. Firstly, to approximate in vivo conditions, HOK cells in the oral mucosa make them an ideal model for studying OLP. The use of LPS was allowed in the present study to reproduce the inflammatory conditions characteristic of OLP. Secondly, to induce inflammation, the LPS allowed for the study of mechanisms leading to inflammation and tissue damage in the context of OLP. Analyzing the HOK response to LPS provides information on the pathophysiological processes occurring in this disease. Thirdly, in response to LPS, HOK can secrete various proinflammatory cytokines that play a key role in the pathogenesis of OLP, which translates into how inflammation contributes to the development of clinical symptoms of OLP.

The HOK cells (CVCL_B404) were isolated from human (Homo Sapiens) oral mucosa. Cell line was obtained from Scien Cell (catalog No. 2610) and incubated in keratinocyte medium (catalog No. 2611; Scien Cell, Carlsbad, CA, USA) supplemented with 1% keratinocyte growth factor plus epithelial growth factor mixture and 1% streptomycin–penicillin solution (Invitrogen; Thermo Fisher Scientist, Inc. (Waltham, MA, USA); catalog No. 15140122) at 37 °C in an atmosphere of 5% CO_2_. Cells from the third passage were used in experiments. To obtain conditions corresponding to the inflammatory state occurring in oral lichen planus, HOK cell culture was used according to the previous studies by X. Ge et al., as described below. HOK cells were grown in 96-well plates at a 5 × 10^3^ cells/well density and cultured overnight at 37 °C in 5% CO_2_. The present study treated HOK cells with LPS to mimic the infection-induced OLP state. In the 3-(4,5-dimethylthiazol-2-yl)-2,5-diphenyltetrazolium bromide (MTT) assay, HOK cells were incubated with a range of concentrations (5, 10, 50, 100, 200, 230, 300, 500, 750 and 1000 µg/mL) of Jantar (JA) and Szafir (SZ) extracts for 48 h for assessment. The extracts were diluted in complete culture medium and added directly to the wells of a 96-well plate, with each well containing 200 µL of medium and a final concentration of the extract as indicated.

### 4.5. Cell Viability Assay

In the next step of the experiment, MTT in the concentration of 0.5 mg/ml (diluted in RPM I1640 without phenol red; Sigma-Aldrich, Poznan, Poland; catalog No. 298-93-1) was added to each well. The plates were incubated for at least two hours, yielding purple MTT formazan crystals in the wells. The crystals’ dissolution followed the incubation with MTT in the acidic isopropanol mixture (isopropanol: HCl, *v/v* 1:0.04). The absorbance of the obtained product was read at 570 nm using a microplate reader. Survival rate was expressed as the percentage of cell survival and calculated from the ratio (A_570_ of treated cells/A_570_ of control cells) × 100%. The experiments were repeated three times.

### 4.6. Quantification of Interleukin-6 (IL-6) and Cyclooxygenase 2 (COX-2) Using Enzyme-Linked Immunosorbent Assay (ELISA)

HOK cells were seeded in 96-well plates at 150,000 cells/ml. The HOK cells were treated with 100 μM of lipopolysaccharide (LPS; trinitrophenol-lipopolysaccharide from E. coli O111:B4, Sigma-Aldrich, Poznan, Poland; catalog No. 93572-42-0) added at the time of seeding. A fresh medium containing the compounds (10 µg/mL) was added after 24 h. After incubation with the studied compounds for 48 h, IL-6 and COX-2 were quantified using IL-6 Human ELISA Kit (ThermoFisher Scientific, Waltham, MA, USA; catalog No. EH2IL6) and Human COX-2 ELISA Kit (Sigma-Aldrich, Poznan, Poland; catalog No. RAB1034-1KT), respectively. ELISA was performed according to the manufacturer’s instructions. Each plate test was repeated three times. Positive control was not included in the present study.

### 4.7. Data Analysis

All of the experiments were repeated three times. Data represent the mean ± standard deviation (SD) of at least three replications. Students’ *t*-test was applied, and *p*-values of less than 0.05 were considered to achieve statistical significance.

## 5. Conclusions

- The extracts from the JA and SZ flax cultivars showed the presence of other phenolic compounds characterized by anti-inflammatory properties.
- The use of Jantarol and Szafir flaxseed extracts showed anti-inflammatory action on oral keratinocyte cell lines. Further clinical studies have to be performed to confirm its action in vivo.
- There was a high in silico affinity of SDG to ERs.
- The research presented concludes the potential use of flaxseed in developing clinically available products.

Limitations: This work did not evaluate the complete structure of the linseed extracts used in the phytotherapy of OLP patients. As a preliminary study, we established a basis for the animal model analysis of the same parameters, which will be the second step of this research.

## Figures and Tables

**Table 1:** Phenolic compounds content in flaxseed extracts of Szafir and Jantarol varieties.

Phenolic Compounds	Contents [mg/100 mL of Defatted Seeds]	
	Szafir (93.14 g of Dry Weight)	Jantarol (92.15 g of Dry Weight)
caffeic acid glucoside	142.10	56.06
coumaric acid glucoside	468.62	133.87
ferulic acid glucoside	566.59	186.85
*p*-coumaric–ferulic glucoside	43.33	29.26
*caffeic-p*-–coumaric glucoside	33.87	23.00
coumaric acid	28.11	9.73
ferulic acid	179.96	31.89
**Total phenolic acids**	**1462.58**	**470.66**
SDG	1436.59	1467.13
**Total phenolic compounds**	**2899.17**	**1937.79**

**Table 2:** Binding energies of analyzed compound to (**a**) ERα and (**b**) ERβ obtained from molecular docking (ΔG_bind_—free energy of binding; ΔE_int_—intermolecular interaction energy; ΔE_1_ = ΔE_vdw_ + ΔE_hbond_ + ΔE_desolv_; ΔE_2_ = ΔE_el_ [kcal/mol]).

**a**	**ΔG_bind_**	**ΔE_int_**	**ΔE_1_**	**ΔE_2_**
SDG	−12.3	−16.1	−16.1	0.0
SECO	−13.3	−20.8	−20.8	0.0
Estradiol	−11.2	−11.2	−11.0	0.0
**b**	**ΔG_bind_**	**ΔE_int_**	**ΔE_1_**	**ΔE_2_**
SDG	−10.95	−14.5	−14.5	0.0
SECO	−11.5	−15.5	−15.5	0.0
Estradiol	−11.9	−12.5	−12.5	0.0

## Data Availability

The data supporting this study’s findings are available from the corresponding author, I.D.I., upon reasonable request.

## Supplementary Materials

The following supporting information can be downloaded at: https://www.mdpi.com/article/10.3390/ijms26125462/s1.

## References

[1] Rosa E.A., Hurtado-Puerto A.M., Falcão D.P., Brietzke A.P., De Almeida Prado Franceschi L.E., Cavalcanti Neto F.F., Tiziane V., Carneiro F.P., Kogawa E.M., Moreno H. (2018). Oral lichen planus and malignant transformation: The role of p16, Ki-67, Bub-3 and SOX4 in assessing precancerous potential. Exp. Ther. Med..

[2] Sen S., Sen S., Dutta A., Abhinandan A., Kumar V., Singh A.K. (2020). Oral manifestation and its management in postmenopausal women: An integrated review. Prz. Menopauzalny.

[3] Lauritano D., Arrica M., Lucchese A., Valente M., Pannone G., Lajolo C., Ninivaggi R., Petruzzi M. (2016). Oral lichen planus clinical characteristics in Italian patients: A retrospective analysis. Head Face Med..

[4] Tsushima F., Sakurai J., Uesugi A., Oikawa Y., Ohsako T., Mochizuki Y., Hirai H., Kayamori K., Harada H. (2021). Malignant transformation of oral lichen planus: A retrospective study of 565 Japanese patients. BMC Oral Health.

[5] Gangeshetty N. (2015). Oral lichen planus: Etiology, pathogenesis, diagnosis, and management. World J. Stomatol..

[6] Lavanya N., Jayanthi P., Rao U., Ranganathan K. (2011). Oral lichen planus: An update on pathogenesis and treatment. J. Oral Maxillofac. Pathol..

[7] Daume L., Kreis C., Bohner L., Jung S., Kleinheinz J. (2021). Clinical characteristics of oral lichen planus and its causal context with dental restorative materials and oral health-related quality of life. BMC Oral Health.

[8] Parlatescu I., Tovaru M., Nicolae C.L., Sfeatcu R., Didilescu A.C. (2020). Oral health-related quality of life in different clinical forms of oral lichen planus. Clin. Oral Investig..

[9] Wardrop R.W., Hailes J., Burger H., Reade P.C. (1989). Oral discomfort at menopause. Oral Surgery, Oral Med. Oral Pathol..

[10] Radwan-Oczko M., Rybińska A., Mierzwicka A., Duś-Ilnicka I. (2024). Salivary Histamine Levels in Patients with Oral Lichen Planus Lesions. Medicina.

[11] Mohan R.P.S., Gupta A., Kamarthi N., Malik S., Goel S., Gupta S. (2017). Incidence of Oral Lichen Planus in Perimenopausal Women: A Cross-sectional Study in Western Uttar Pradesh Population. J. Mid-Life Health.

[12] Pertyński T., Stachowiak G. (2006). Menopauza—Fakty i kontrowersje. Endokrynol. Pol..

[13] Heldring N., Pike A., Andersson S., Matthews J., Cheng G., Hartman J., Tujague M., Ström A., Treuter E., Warner M. (2007). Estrogen receptors: How do they signal and what are their targets. Physiol. Rev..

[14] Dietrich W., Haitel A., Holzer G., Huber J.C., Kolbus A., Tschugguel W. (2006). Estrogen Receptor-β Is the Predominant Estrogen Receptor Subtype in Normal Human Synovia. J. Soc. Gynecol. Investig..

[15] Paterni I., Granchi C., Katzenellenbogen J.A., Minutolo F. (2011). Estrogen Receptors Alpha and Beta Subtype—Selective Ligands and Clinical Potential. Steroids.

[16] Alshahrani I., Hameed M.S., Syed S., Amanullah M., Togoo R.A., Kaleem S. (2019). Changes in essential salivary parameters in patients undergoing fixed orthodontic treatment: A longitudinal study. Niger. J. Clin. Pract..

[17] Chi A.C., Neville B.W., Krayer J.W., Gonsalves W.C. (2010). Oral manifestations of systemic disease. Am. Fam. Physician.

[18] Basch E., Bent S., Collins J., Dacey C., Hammerness P., Harrison M., Smith M., Szapary P., Ulbricht C., Vora M. (2007). Flax and flaxseed oil (*Linum usitatissimum*): A review by the Natural Standard Research Collaboration. J. Soc. Integr. Oncol..

[19] Morales-Bozo I., Ortega-Pinto A., Rojas Alcayaga G., Aitken Saavedra J.P., Salinas Flores O., Lefimil Puente C., Lozano Moraga C., Manríquez Urbina J.M., Urzúa Orellana B. (2017). Evaluation of the effectiveness of a chamomile (*Matricaria chamomilla*) and linseed (*Linum usitatissimum*) saliva substitute in the relief of xerostomia in elders. Gerodontology.

[20] Andersson G., Johansson G., Attström R., Edwardsson S., Glantz P.O., Larsson K. (1995). Comparison of the effect of the linseed extract Salinum and a methyl cellulose preparation on the symptoms of dry mouth. Gerodontology.

[21] Ameri A., Heydarirad G., Jafari J.M., Ghobadi A., Rezaeizadeh H., Choopani R. (2015). Medicinal plants contain mucilage used in traditional Persian medicine (TPM). Pharm. Biol..

[22] Aitken-Saavedra J., Chaves Tarquinio S.B., De Oliveira Da Rosa W.L., Fernandes Da Silva A., Almeida MacHado B.M.E., Santos Castro I., Oliveira Wennesheimer A., Morales-Bozo I., Uchoa Vasconcelos A.C., Neutzling Gomes A.P. (2020). Effect of a Homemade Salivary Substitute Prepared Using Chamomile *Matricaria chamomilla* L. Flower and Flax *Linum usitatissimum* L. Seed to Relieve Primary Burning Mouth Syndrome: A Preliminary Report. J. Altern. Complement. Med..

[23] Kręgielczak A., Łukaszewska-Kuska M., Mania-Końsko A., Dorocka-Bobkowska B. (2023). Flaxseed (*Linum usitatissimum*), Chamomile (*Matricariae flos*) and Marshmallow (*Althaea officinalis*) Mouth Rinses in the Therapy of Oral Mucosa Diseases—A Review. J. Nat. Fibers.

[24] Dzuvor C.K.O., Taylor J.T., Acquah C., Pan S., Agyei D. (2018). Bioprocessing of functional ingredients from flaxseed. Molecules.

[25] Morris D.H. (2007). Backgrounder on Lignans. Flax: A Health Nutrition Primer.

[26] Toure A., Xu X. (2010). Flaxseed Lignans: Source, Biosynthesis, Metabolism, Antioxidant Activity, Bio-Active Components, and Health Benefits. Compr. Rev. Food Sci. Food Saf..

[27] Strandås C., Kamal-Eldin A., Andersson R., Åman P. (2008). Composition and properties of flaxseed phenolic oligomers. Food Chem..

[28] Lehraiki A., Attoumbre J., Matifat F. (2010). Extraction of Lignans from Flaxseed and Evaluation of Their Biological Effects on Breast Cancer MCF-7 and MDA-MB-231 Cell Lines Abdelali. J. Med. Food.

[29] Sainvitu P., Nott K., Richard G., Blecker C., Jérôme C., Wathelet J.P., Paquot M., Deleu M. (2012). Structure, properties and obtention routes of flaxseed lignan secoisolariciresinol: A review. Biotechnol. Agron. Soc. Environ..

[30] Powers C.N., Setzer W.N. (2015). A molecular docking study of phytochemical estrogen mimics from dietary herbal supplements. Silico Pharmacol..

[31] Martynowicz H., Jodkowska A., Poręba R., Mazur G., Więckiewicz M. (2021). Demographic, clinical, laboratory, and genetic risk factors associated with COVID-19 severity in adults: A narrative review. Dent. Med. Probl..

[32] Orzechowska-Wylęgała B.E., Wylęgała A.A., Zalejska-Fiolka J., Czuba Z., Toborek M. (2024). Pro-inflammatory cytokines and antioxidative enzymes as salivary biomarkers of dentofacial infections in children. Dent. Med. Probl..

[33] Kerstjens A., De Winter H. (2022). LEADD: Lamarckian evolutionary algorithm for de novo drug design. J. Cheminform..

[34] Chen D., Menche G., Power T.D., Sower L., Peterson J.W., Schein C.H. (2007). Accounting for ligand-bound metal ions in docking small molecules on adenylyl cyclase toxins. Proteins.

[35] Duś-Ilnicka I. (2024). Neglected area of oral cancer: A word about the International Agency for Research on Cancer (IARC) “Handbook of Oral Cancer Prevention”. Dent. Med. Probl..

[36] Soria A., Agbo-Godeau S., Taïeb A., Francès C. (2008). Treatment of refractory oral erosive lichen planus with topical rapamycin: 7 Cases. Dermatology.

[37] Maloth K.N., Sunitha K., Boyapati R., Shravan Kumar D.R. (2014). Bullous lichen planus treated with oral minipulse therapy: A rare case report. J. Clin. Diagn. Res..

[38] Kazanowska-Dygdała M., Duś I., Radwan-Oczko M. (2016). The presence of Helicobacter pylori in oral cavities of patients with leukoplakia and oral lichen planus. J. Appl. Oral Sci..

[39] Agha-Hosseini F., Mirzaii-Dizgah I., Mikaili S., Abdollahi M. (2009). Increased salivary lipid peroxidation in human subjects with oral lichen planus. Int. J. Dent. Hyg..

[40] Eliasson L., Carlén A., Laine M., Birkhed D. (2003). Minor gland and whole saliva in postmenopausal women using a low potency oestrogen (oestriol). Arch. Oral Biol..

[41] Leimola-Virtanen R., Salo T., Toikkanen S., Pulkkinen J., Syrjänen S. (2000). Expression of estrogen receptor (ER) in oral mucosa and salivary glands. Maturitas.

[42] Parikh M., Maddaford T.G., Austria J.A., Aliani M., Netticadan T., Pierce G.N. (2019). Dietary Flaxseed as a Strategy for Improving Human Health. Nutrients.

[43] EMA (2005). European Union Herbal Monograph on Linum usitatissimum L., Semen.

[44] Barbary O.M., El-Sohaimy S.A., El-Saadani M.A., Zeitoun A.M. (2010). Antioxidant, Antimicrobial and Anti-HCV Activities of Lignan Extracted from Flaxseed. Res. J. Agric. Biol. Sci..

[45] Chen M.N., Lin C.C., Liu C.F. (2015). Efficacy of phytoestrogens for menopausal symptoms: A meta-analysis and systematic review. Climacteric.

[46] Kuiper G.G.J.M., Lemmen J.G., Carlsson B., Corton J.C., Safe S.H., Van Der Saag P.T., Van Der Burg B., Gustafsson J.Å. (1998). Interaction of estrogenic chemicals and phytoestrogens with estrogen receptor β. Endocrinology.

[47] Yaşar P., Ayaz G., User S.D., Güpür G., Muyan M. (2017). Molecular mechanism of estrogen-estrogen receptor signaling. Reprod. Med. Biol..

[48] Tomasi J., Mennucci B., Cammi R. (2005). Quantum Mechanical Continuum Solvation Models. Chem. Rev..

[49] Souza P.C.T., Textor L.C., Melo D.C., Nascimento A.S., Skaf M.S., Polikarpov I. (2017). An alternative conformation of ERβ bound to estradiol reveals H12 in a stable antagonist position. Sci. Rep..

[50] Sharma P., Joshi T., Joshi T., Chandra S., Tamta S. (2020). In silico screening of potential antidiabetic phytochemicals from Phyllanthus emblica against therapeutic targets of type 2 diabetes. J. Ethnopharmacol..

[51] Sayed A.A., Elfiky A.A. (2018). In silico estrogen-like activity and in vivo osteoclastogenesis inhibitory effect of Cicer arietinum extract. Cell. Mol. Biol..

[52] Zeng W., Takashima K., Tang Q., Zou X., Ojiro R., Ozawa S., Jin M., Ando Y., Yoshida T., Shibutani M. (2023). Natural antioxidant formula ameliorates lipopolysaccharide-induced impairment of hippocampal neurogenesis and contextual fear memory through suppression of neuroinflammation in rats. J. Chem. Neuroanat..

[53] Baby T.K., Bindhu P.R., Pillai R.K., Jayanthi P. (2022). Immunohistochemical expression of cyclooxygenase-2 in oral lichen planus and normal oral mucosa. Indian J. Pathol. Microbiol..

[54] Beristain-Colorado M.D.P., Castro-Gutiérrez M.E.M., Torres-Rosas R., Vargas-Treviño M., Moreno-Rodríguez A., Fuentes-Mascorro G., Argueta-Figueroa L. (2024). Application of neural networks for the detection of oral cancer: A systematic review. Dent. Med. Probl..

[55] Stewart J.J.P. (2007). Optimization of parameters for semiempirical methods V: Modification of NDDO approximations and application to 70 elements. J. Mol. Model..

[56] Morris G.M., Huey R., Lindstrom W., Sanner M.F., Belew R.K., Goodsell D.S., Olson A.J. (2009). AutoDock4 and AutoDockTools4: Automated docking with selective receptor flexibility. J. Comput. Chem..

[57] Tanenbaum D.M., Wang Y., Williams S.P., Sigler P.B. (1998). Crystallographic comparison of the estrogen and progesterone receptor’s ligand binding domains. Proc. Natl. Acad. Sci. USA.

[58] Möcklinghoff S., Rose R., Carraz M., Visser A., Ottmann C., Brunsveld L. (2010). Synthesis and crystal structure of a phosphorylated estrogen receptor ligand binding domain. Chembiochem.

[59] Velázquez-Libera J.L., Durán-Verdugo F., Valdés-Jiménez A., Núñez-Vivanco G., Caballero J. (2020). LigRMSD: A web server for automatic structure matching and RMSD calculations among identical and similar compounds in protein-ligand docking. Bioinformatics.

[60] Szczęśniak-Sięga B.M., Wiatrak B., Czyżnikowska Ż., Janczak J., Wiglusz R.J., Maniewska J. (2021). Synthesis and biological evaluation as well as in silico studies of arylpiperazine-1,2-benzothiazine derivatives as novel anti-inflammatory agents. Bioorg. Chem..

[61] Janek T., Czyżnikowska Ż., Łukaszewicz M., Gałęzowska J. (2019). The effect of Pseudomonas fluorescens biosurfactant pseudofactin II on the conformational changes of bovine serum albumin: Pharmaceutical and biomedical applications. J. Mol. Liq..

[62] Zeitoun A.M., Preisner M., Kulma A., Dymińska L., Hanuza J., Starzycki M., Szopa J. (2014). Does biopolymers composition in seeds contribute to the flax resistance against the Fusarium infection?. Biotechnol. Prog..

