# Supplementary material for: Flaxseed Extracts Impact the Cellular Structure of a Keratinocyte Model for Oral Lichen Planus—A Preliminary Study
Source: Int J Mol Sci. 2025 Jun 6;26(12):5462. doi: 10.3390/ijms26125462 (PMC12192927; doi:10.3390/ijms26125462)
Supplement: Supplementary file 1 [file ijms-26-05462-s001.zip › ijms-3598467-supplementary.pdf]

Supplementary table S1. Retention time and UV-VIS spectra of phenol substrates identified in the Jantarol and Szafir flaxseed varieties extracts.

Compund	***RT [min]***	***UV-VIS spectra***
glucoside of caffeic acid	1,08	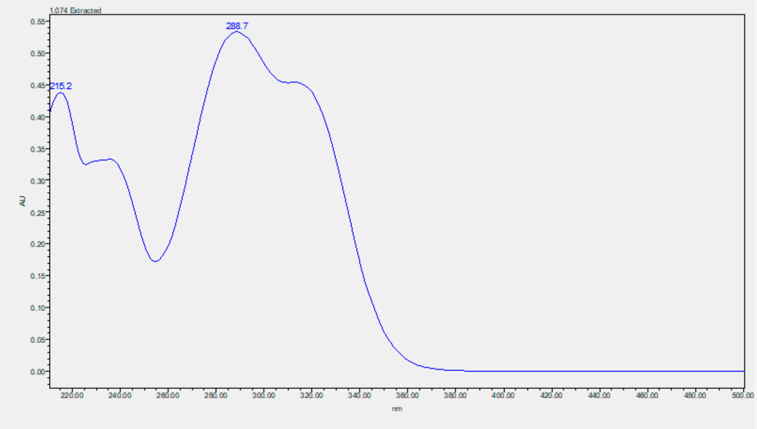
cumaric acid glycoside	1,149	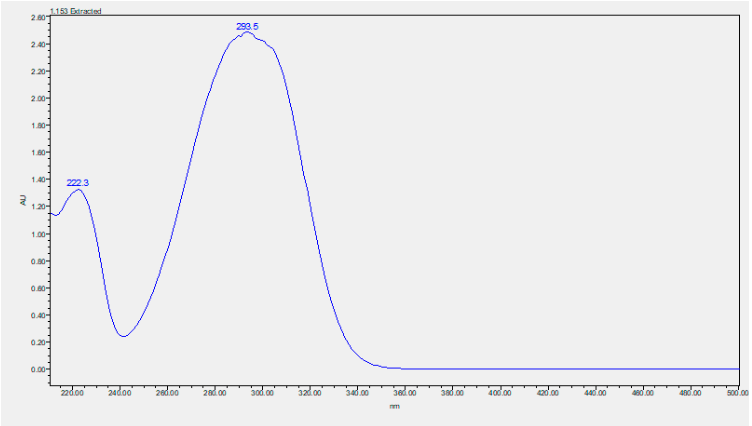
ferulic acid glucoside (2))	1,42	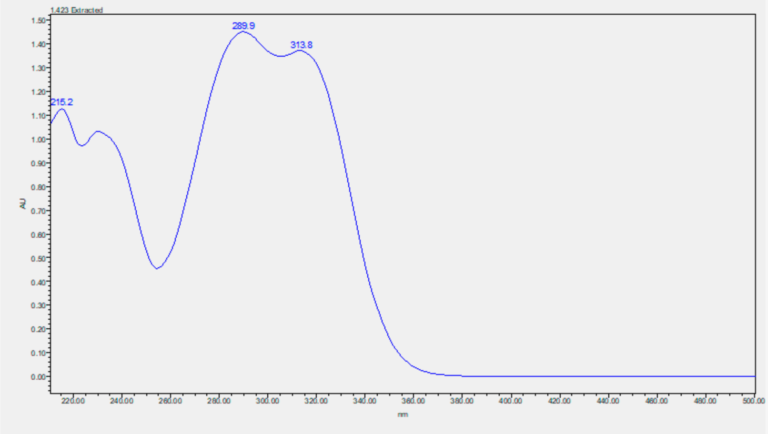

cumaric feruloyl glycoside	2,86	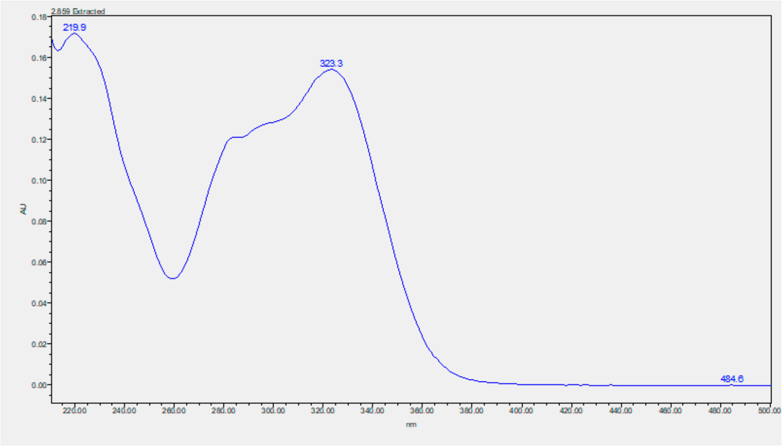
coffeic-coumarin glycoside	2,95	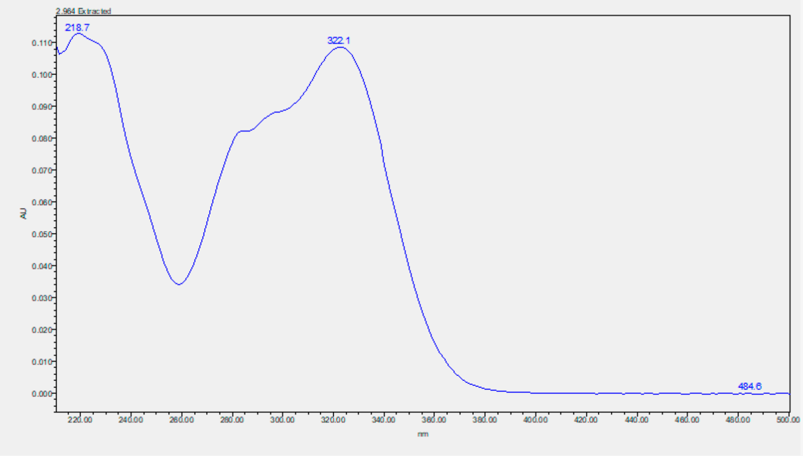
ferulic acid glucoside (1))	3,27	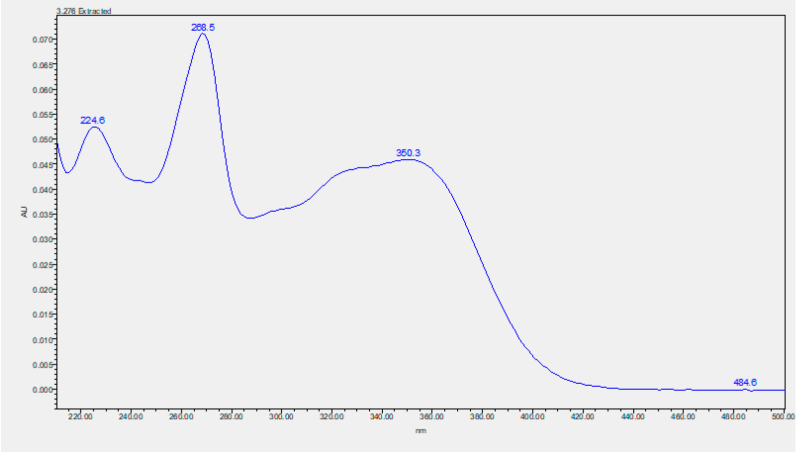

cumaric acid	3,78	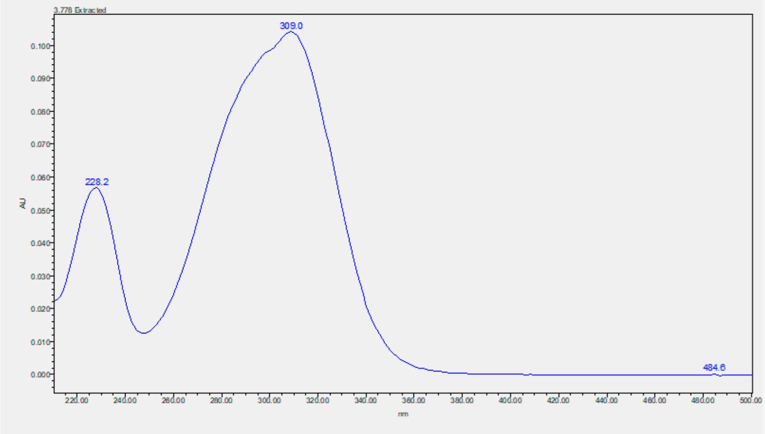
SDG	**4,37**	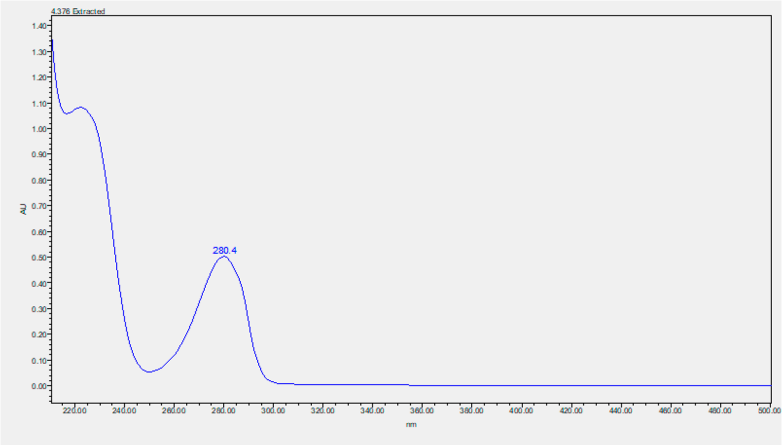
ferulic acid	4,44	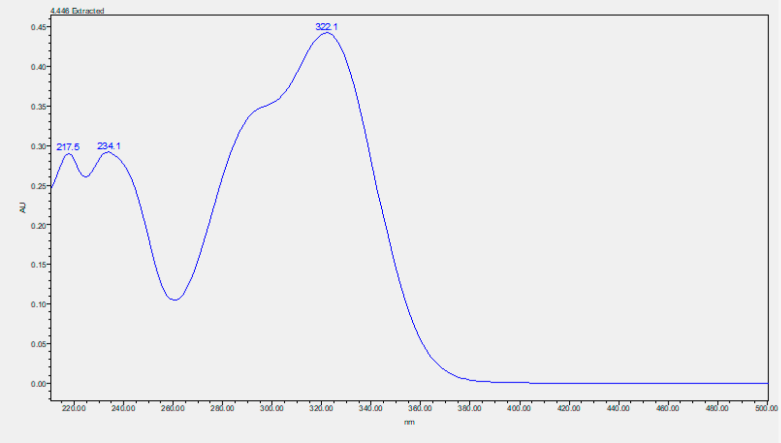

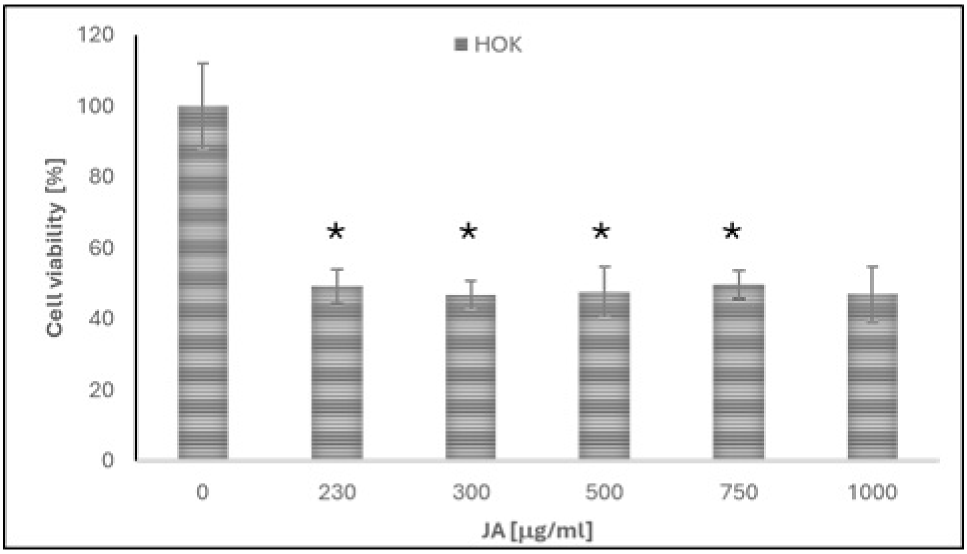

Figure S1. Additional data provided IC50 value with the use of Jantarol cultivar.

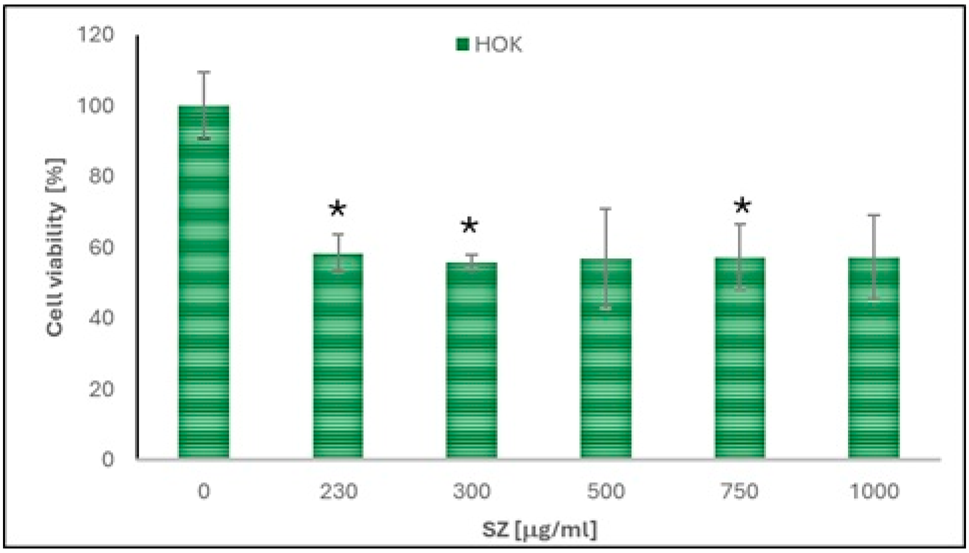

Figure S2. Additional data provided IC50 value with the use of Szafir cultivar.
